# Supplementary material for: The Capacity of Long-Term In Vitro Proliferation of Acute Myeloid Leukemia Cells Supported Only by Exogenous Cytokines Is Associated with a Patient Subset with Adverse Outcome
Source: Cancers (Basel). 2019 Jan 10;11(1):73. doi: 10.3390/cancers11010073 (PMC6356272; doi:10.3390/cancers11010073)
Supplement: Supplementary file 1 [file cancers-11-00073-s001.pdf]

cancers MDPI

The Capacity of Long-Term in Vitro Proliferation of Acute Myeloid Leukemia Cells Supported Only by Exogenous Cytokines Is Associated with a Patient Subset with Adverse Outcome

Annette K. Brenner, Elise Aasebø, Maria Hernandez-Valladares, Frode Selheim, Frode Berven, Ida-Sofie Grønningsæter, Sushma Bartaula-Brevik and Øystein Bruserud

Supplementary Material

Table S1. Detailed information about the 68 AML patients included in the study.

ID	Gender	Age	Etiology	# of blasts (10^9/L)	FAB	Cytogenetics	Mutations	CD34	Viability (%) 48 h	Proliferation (cpm)	Cytokine secretion	Viable cells (10^6) 5 weeks	Colonies	Change in phenotype
1	M	42	*de novo*	241	M2	normal	*Flt3*	pos	31.0	3848	low	0.24	7	yes
2	M	82	MF	12.4	M2	t(9;22)	wt	pos	81.6	74,686	low	1.43	969	yes
3	F	49	CML/relapse	149	M2	complex	n.d.	pos	26.2	3472	low	0.08	n.d.	no
4	M	33	*de novo*	62.0	M2	normal	wt	pos	67.5	6206	low	0.08	6.5	no
5	M	71	relapse	91.0	M4	normal	*NPM1*	pos	63.5	21,331	low	0.17	n.d.	yes
6	M	83	*de novo*	109	M1	n.d.	wt	pos	19.1	8764	low	1.65	693	no
7	F	77	MDS	26.4	M1	normal	wt	pos	89.4	53,799	high	3.43	2746	no
8	M	46	*de novo*	26.9	M1	normal	*NPM1*	n.d.	n.d.	3472	low	1.56	n.d.	no
9	M	68	MF	50.8	M4	normal	D835	pos	69.4	1640	low	0.08	n.d.	no
10	F	71	MDS/relapse	238	n.d.	del(12)	n.d.	pos	n.d.	97,725	high	14.3	286	yes
11	F	86	*de novo*	15.0	M1	normal	n.d.	pos	45.5	12,664	high	0.18	n.d.	yes
12	M	66	*de novo*	33.0	M5	t(16;16)	wt	pos	67.8	5477	high	3.30	396	yes
13	F	55	*de novo*	145	M2	normal	*Flt3/NMP1*	neg	86.0	13,454	low	0.04	n.d.	no
14	M	71	*de novo*	126	M4/5	n.d.	n.d.	n.d.	n.d.	n.d.	high	0.62	n.d.	yes
15	F	18	*de novo*	53.6	M4	inv(16)	wt	pos	61.3	12,713	high	0.05	1.5	yes
16	F	77	MDS	55.9	M1/2	normal	*Flt3/NMP1*	n.d.	69.8	11,102	high	0.59	285	no
17	M	71	*de novo*	2.3	n.d.	n.d.	n.d.	n.d.	n.d.	n.d.	low	0.01	n.d.	no
18	F	46	*de novo*	32.5	M1	inv(16)	wt	pos	68.1	50,873	high	0.47	n.d.	yes
19	F	75	MF	41.0	M2	n.d.	n.d.	pos	22.4	52,071	high	0.25	n.d.	yes
20	F	45	chemo	65.0	M4	normal	*NPM1*	neg	3.8	< 1000	low	0	n.d.	no
21	F	78	*de novo*	153	M1	normal	*Flt3/NMP1*	neg	n.d.	16,207	high	53.5	29,959	yes
22	F	42	*de novo*	40.4	M5	normal	*NPM1*	neg	18.9	< 1000	high	0.07	12.5	yes
23	M	60	MDS	108	M2	normal	n.d.	pos	74.4	6590	low	0.04	n.d.	no
24	M	60	*de novo*	22.6	M4	del(9)	*Flt3*	pos	42.4	8022	low	0.90	18	yes
25	F	49	*de novo*	126	M5	normal	*Flt3/NMP1*	neg	50.8	1311	high	1.16	4617	yes
26	M	35	*de novo*	241	M2	normal	wt	pos	80.3	97,008	high	0.79	n.d.	yes
27	M	76	MDS	12.4	n.d.	normal	n.d.	pos	55.1	10,901	low	3.06	n.d.	yes
28	M	72	MDS	38.0	M1	complex	wt	pos	60.6	2117	low	0.10	24	no

29	F	71	MDS	19.6	n.d.	t(1;5), t(2;3)	n.d.	pos	60.6	18,044	high	0.39	47	yes
30	F	33	*de novo*	25.6	M1	t(8;21)	wt	pos	53.7	1533	low	0.12	3	yes
31	M	82	*de novo*	40.8	n.d.	+8	wt	pos	40.7	< 1000	high	0.10	n.d.	no
32	M	58	*de novo*	70.7	M5	normal	wt	pos	10.6	< 1000	high	0.01	16	yes
33	F	55	*de novo*	103	M5	normal	*Flt3/NPM1*	neg	26.5	10,534	high	1.70	34	yes
34	M	65	*de novo*	21.0	M4	normal	*NPM1*	neg	37.8	1693	high	2.25	180	no
35	M	64	*de novo*	88.5	M5	normal	*NPM1*	neg	66.3	2106	high	0.13	n.d.	yes
36	M	48	*de novo*	48.9	M5	normal	*Flt3/NPM1*	neg	26.7	16,980	high	1.80	n.d.	yes
37	M	78	*de novo*	56.1	M1	complex	n.d.	pos	54.4	10,032	low	0.36	65	yes
38	M	36	*de novo*	241	M4	inv(16)	wt	pos	n.d.	n.d.	high	0.06	n.d.	yes
39	F	92	CLL	61.5	M1	n.d.	n.d.	neg	n.d.	1141	low	2.02	1455	no
40	F	59	*de novo*	21.4	M4	normal	*Flt3/NPM1*	neg	69.5	10,711	low	10.7	35,050	no
41	M	61	*de novo*	5.5	M4	normal	wt	neg	n.d.	6568	high	10.4	8972	yes
42	F	57	*de novo*	65.4	M4	inv(16)	wt	pos	67.7	20,371	high	1.07	n.d.	yes
43	M	20	*de novo*	168	M2	normal	*Flt3*	pos	n.d.	20,767	high	32.6	8141	yes
44	F	64	*de novo*	64.5	M2	normal	*Flt3/NPM1*	neg	2.6	< 1000	high	0	n.d.	no
45	F	29	*de novo*	255	M5	normal	*Flt3*/D835	pos	54.2	102,303	high	1.15	n.d.	yes
46	F	63	*de novo*	61.8	M1	normal	wt	pos	77.0	121,748	high	0.27	n.d.	no
47	F	61	MDS/relapse	4.4	M1	complex	wt	neg	n.d.	n.d.	high	1.15	483	yes
48	F	80	PV	32.9	M4/5	complex	wt	n.d.	n.d.	n.d.	high	0.10	3	yes
49	M	76	CMML	12.7	M5	del(12), -7	n.d.	pos	31.5	173,197	high	0.40	n.d.	yes
50	F	74	*de novo*	30.0	M4	t(8;21)	*Flt3*	pos	30.3	4462	low	0.77	316	no
51	M	74	*de novo*	27.9	M1	complex	wt	pos	63.8	136,388	high	0.06	n.d.	no
52	M	78	*de novo*	220	M0	complex	wt	neg	35.1	< 1000	low	0.60	96	no
53	F	68	*de novo*	214	M5	normal	*NPM1*	neg	63.9	1220	low	0.30	474	no
54	M	65	*de novo*	101	M5	complex	*NPM1*	n.d.	35.1	< 1000	high	1.04	21	yes
55	F	64	relapse	55.0	M4	complex	wt	pos	34.6	34,137	high	1.37	n.d.	no
56	M	64	relapse	48.0	M2	normal	*NPM1*	neg	14.5	11,636	low	2.00	n.d.	no
57	F	81	MDS	103	M1	normal	wt	pos	n.d.	n.d.	low	1.02	n.d.	no
58	M	72	MDS	105	M4	normal	n.d.	neg	52.6	12,919	high	0.88	n.d.	yes
59	M	77	*de novo*	148	M2	complex	*Flt3*	pos	66.1	7720	low	2.89	3810	yes

60	F	55	*de novo*	67.3	M1	normal	*Flt3/NPM1*	pos	42.9	1062	low	1.36	843	no
61	M	76	*de novo*	20.6	M0	normal	wt	pos	50.9	9407	low	0.07	98	no
62	M	62	*de novo*	2.0	M4	+8	wt	pos	74.6	81,412	high	4.37	5329	no
63	F	87	*de novo*	102	M0	del(5)	wt	pos	47.5	< 1000	low	0.94	1278	no
64	M	59	CMML/relapse	77.0	M5	del(20); +8	*Flt3*	pos	41.3	7344	low	1.24	6770	yes
65	F	71	*de novo*	40.9	M0	normal	*NPM1*	neg	54.1	6791	low	11.1	333	no
66	F	46	*de novo*	87.0	M2	inv(13)	wt	pos	88.3	62,581	low	1.87	n.d.	no
67	F	77	*de novo*	168	M1	n.d.	*NPM1*	neg	46.5	9839	low	11.3	7230	yes
68	M	19	*de novo*	12.1	M5	normal	wt	neg	71.9	5296	high	0.20	n.d.	yes

	**Meaning of color codes**
(yellow)	Survival data
(orange)	Survival data and microarray
(red)	Microarray
(green)	Survival data and proteomics
(purple)	Microarray and proteomics
(blue)	Proteomics
(grey)	Survival data, microarray and proteomics

Table S2. Week5/week 1 secretion ratios for 19 soluble mediators. Values are provided for all patients (left), and then divided into groups with and without detectable colonies. Significantly increased levels (Mann-Whitney *U*-test) are highlighted.

	**All (n = 62) ***		**Colonies (n = 33)**		**No colonies (n = 29)**		***p*-values**
	**Median and range**		**Median and range**		**Median and range**		
CXCL1	1.04	0.01–1831	1.05	0.01–12	1.02	0.01–1831	NS
CXCL5	2.30	0.01–22,488	2.38	0.01–22,488	1.96	0.01–959	NS
CXCL8	3.06	0.01–70	2.78	0.10–70	5.46	0.01–54	NS
CXCL10	1.24	0.10–157	1.25	0.20–24	1.11	0.10–157	NS
CCL2	6.64	0.01–400	7.40	0.28–400	4.70	0.01–192	NS
CCL3	1.14	0.15–239	1.14	0.54–239	1.14	0.15–6.87	NS
CCL4	1.77	0.09–104	1.75	0.09–104	1.86	0.16–49	NS
CCL5	1.00	0.01–5327	1.00	0.01–5327	1.00	0.01–44	NS
IL-1β	1.09	0.01–30	1.09	0.01–22	1.17	0.01–30	NS
IL-1RA	4.67	0.01–358	**8.31**	**0.45–207**	2.71	0.01–358	**0.014**
IL-6	1.02	0.01–249	0.94	0.01–176	1.41	0.01–249	NS
HGF	2.65	0.02–108	**3.77**	**0.37–108**	2.75	0.02–7.02	**0.004**
G-CSF	1.81	0.01–230	1.88	0.05–230	1.76	0.01–128	NS
Cystatin C	4.14	0.04–46	4.61	1.08–30	3.06	0.04–46	NS
Serpin E1	1.98	0.02–73	1.80	0.05–35	2.19	0.02–73	NS
TNFα	2.02	0.01–1012	2.09	0.08–1012	1.89	0.01–783	NS
MMP-1	3.92	0.02–465	4.07	0.37–90	3.76	0.02–465	NS
MMP-2	3.84	0.04–49	5.25	0.04–32	3.13	0.11–49	NS
MMP-9	2.11	0.01–392	2.28	0.01–392	1.49	0.04–122	NS

* Due to technical reasons, the week 5 values for six patients are missing.

Table S3. HGF, IL-1RA and MMP-9 levels from long-time suspension cultures of primary human AML cells; a comparison of the supernatant levels after 1 and 5 weeks of suspension culture. Leukemic cells derived from 62 patients were included in this part of the study; because the week 5 values for six patients are missing, their week 1 values were censored for better comparison. All results are presented as the median levels (ng/mL) after one 1 (w1) and five (w5) weeks of suspension culture. Significantly increased values (Mann-Whitney *U*-test, $p \leq 0.01$) are highlighted.

Mediator		FAB classification		*p*-values	Phenotypic alteration during culture		*p*-values	Colony formation after long-term suspension culture		*p*-values
		**M0-2 (n=30)**	**M4/5 (n=28)**		**No (n=29)**	**Yes (n=33)**		**No (n=29)**	**Yes (n=33)**	
HGF	w1	0.20 (0.01–7.50)	0.16 (0.01–1.99)	0.018	0.06 (0.01–7.06)	**0.33 (0.04–7.50)**	**0.005**	0.21 (0.01–3.41)	0.17 (0.01–7.50)	0.048
	w5	**1.01 (0.01–4.99)**	0.43 (0.01–7.84)	**0.001**	0.20 (0.01–7.84)	**1.01 (0.06–4.19)**	**0.001**	0.47 (0.01–4.19)	**1.26 (0.02–7.84)**	**<0.001**
IL-1RA	w1	0.43 (0.02–18.66)	**1.45 (0.01–5.61)**	**<0.0001**	0.28 (0.01–18.66)	**1.47 (0.07–5.20)**	**0.001**	**1.20 (0.01–18.66)**	0.57 (0.02–5.61)	**0.007**
	w5	**6.86 (0.01–54.49)**	2.77 (0.01–30.01)	**0.002**	2.69 (0.01–54.49)	**4.88 (0.24–30.01)**	**<0.0001**	2.63 (0.01–14.29)	**7.68 (0.24–54.49)**	**<0.0001**
MMP-9	w1	1.03 (0.01–47.53)	**13.64 (0.01–105.2)**	**0.002**	1.03 (0.01–18.83)	**14.04 (0.12–105.2)**	**<0.001**	3.33 (0.01–105.2)	1.93 (0.01–100.1)	NS
	w5	3.92 (0.01–105.6)	6.79 (0.01–110.3)	NS	4.04 (0.01–105.6)	6.89 (0.01–110.4)	NS	2.79 (0.01–101.9)	6.64 (0.01–110.4)	0.039

Table S4. Differences in the secretion ratios (median and range, in pg/mL) for 19 mediators depending on morphology according to FAB (left) and morphological changes during suspension culture (right). Significantly increased values (Mann-Whitney *U*-test, $p \leq 0.01$) are highlighted.

	FAB classification of primary sample		*p*-values	Altered phenotype during suspension culture		*p*-values
	**M0-2 (n = 30)**	**M4/5 (n = 28)**		**No (n = 29)**	**Yes (n = 33)**	
CCL2	399 (39–30,332)	2391 (52–36,371)	0.018	337 (n.d.–30,332)	**1630 (52–36,371)**	**0.002**
	4637 (48–38,864)	**29,103 (142–41,337)**	**0.007**	2598 (48–32,645)	**26,455 (718–41,337)**	**<0.0001**
CCL3	822 (716–1315)	861 (595–10,207)	0.011	817 (129–1315)	844 (595–10,207)	0.034
	990 (675–15,218)	956 (774–201,644)	NS	901 (675–15,218)	1000 (710–201,644)	NS
CCL4	315 (n.d.–826)	530 (n.d.–8559)	0.019	294 (n.d.–826)	**445 (n.d.–8559)**	**<0.001**
	698 (n.d.–8771)	697 (n.d.–119,899)	NS	557 (n.d.–8771)	759 (289–119,899)	0.022
CCL5	27 (n.d.–205)	16 (n.d.–366)	NS	18 (n.d.–90)	33 (n.d.–366)	NS
	10 (n.d.–4282)	7.2 (n.d.–12,608)	NS	9.1 (n.d.–4282)	11 (n.d.–12,608)	NS
CXCL1	190 (4.0–947)	**210 (150–94,437)**	**0.009**	199 (n.d.–504)	202 (150–94,437)	NS
	217 (177–1771)	258 (154–30,381)	NS	213 (177–1771)	236 (138–30,381)	NS
CXCL5	71 (n.d.–4571)	**1127 (37–70,000)**	**0.003**	63 (n.d.–4571)	**936 (n.d.–70,000)**	**<0.001**
	381 (n.d.–68,762)	**1443 (79–70,000)**	**0.008**	222 (n.d.–68,762)	**592 (79–70,000)**	**<0.001**
CXCL8	335 (27–9000)	1512 (23–9000)	0.012	287 (11–9000)	**1158 (27–9000)**	**<0.001**
	9000 (0.7–9000)	9000 (265–9000)	NS	1788 (0.7–9000)	9000 (265–9000)	NS
CXCL10	44 (5.4–4742)	81 (35–6757)	0.038	43 (2.9–461)	**76 (35–6757)**	**0.001**
	73 (35–6826)	119 (36–4449)	NS	59 (35–6826)	**148 (36–5029)**	**0.006**

Cystatin C	30,291 (1343–264,559)	58,427 (1231–156,589)	NS	27,120 (n.d.–198,186)	**47,018 (6284–264,559)**	**0.002**
	174,708 (n.d.–1190,000)	235,707 (1652–1200,000)	NS	113,932 (n.d.–424,791)	**202,186 (18,648–1200,000)**	**0.010**
G-CSF	5.2 (n.d.–315)	11 (n.d.–6606)	NS	5.2 (n.d.–315)	**9.8 (n.d.–6606)**	**0.005**
	11 (n.d.–602)	15 (n.d.–417)	NS	6.2 (n.d.–602)	**16 (n.d.–417)**	**0.001**
HGF	198 (3.4–7497)	178 (n.d.–1988)	NS	67 (n.d.–7061)	**329 (36–7497)**	**0.001**
	1012 (6.6–4991)	468 (2.3–7841)	NS	228 (2.3– 7841)	1013 (59–4188)	0.044
IL-1β	2.0 (n.d.–8.3)	**4.4 (n.d.–1352)**	**0.007**	1.0 (n.d.–8.3)	**3.6 (n.d.–1352)**	**0.001**
	3.8 (n.d.–53)	5.2 (n.d.–32)	NS	2.4 (n.d.–53)	**6.4 (n.d.–32)**	**0.001**
IL-1RA	432 (24–18,665)	1504 (n.d.–5612)	0.023	288 (n.d.–18,665)	**1473 (67–5197)**	**<0.001**
	6860 (n.d.–54,485)	2848 (n.d.–55,000)	NS	3037 (n.d.–54,485)	4879 (241–55,000)	NS
IL-6	6.3 (0.9–1100)	**24 (1.0–33,519)**	**0.007**	4.4 (0.4–1100)	**21 (1.6–33,519)**	**<0.001**
	11 (1.2–1100)	63 (1.4–7334)	0.018	10 (1.2–1100)	**29 (1.4–7334)**	**0.006**
MMP-1	105 (33–8100)	**160 (78–6637)**	**0.009**	101 (n.d.–8100)	**134 (78–6637)**	**0.007**
	563 (75–8100)	578 (100–8100)	NS	474 (75–8100)	664 (100–8100)	NS
MMP-2	4614 (591–66,836)	7301 (638–24,981)	NS	3759 (n.d.–66,836)	8080 (638–64,768)	0.012
	31,323 (438–90,000)	15,010 (323–84,224)	NS	24,642 (323–90,000)	32,004 (1969–68,425)	NS
MMP-9	1029 (n.d.–47,530)	**14,039 (n.d.–105,195)**	**<0.0001**	1005 (n.d.–18,832)	**14,039 (124–105,195)**	**<0.0001**
	3923 (8.5–105,608)	6940 (n.d.–110,357)	NS	4045 (8.5–105,608)	6836 (n.d.–110,357)	NS
Serpin E1	403 (72–5431)	441 (16–10,294)	NS	374 (16–5431)	503 (47–10,294)	0.016
	887 (26–11,000)	697 (47–10,685)	NS	452 (26–11,000)	1126 (82–11,000)	0.018
TNFα	2.5 (n.d.–245)	**18 (n.d.–2826)**	**<0.001**	2.5 (n.d.–245)	**16 (0.1–2826)**	**<0.0001**
	14 (n.d.–168)	29 (1.6–237)	NS	7.4 (n.d.–168)	**29 (3.3–237)**	**<0.0001**

Table S5. Correlation between cytokine levels and cell or colony number. Only 5/19 cytokines showed significant correlation (*r*-value≥ 0.20) between secretion levels and cell number, and only secretion of MMP-2 was correlated with colony number.

**Mediator**	**Cytokine levels vs.**	
	**Colony number**	**Cell viability**
MMP-2	0.227	0.366
HGF	NS	0.349
Cystatin C	NS	0.313
IL-1RA	NS	0.263
CXCL8	NS	0.216

Table S6. Single genes identified in the comparison of global gene expression profiles for patients with and without at least 0.01% long-term proliferating cells. A total of 14 annotated genes were identified, thereof 12 with a known function. Genes with a positive t-score are overexpressed in the patient group with many clonogenic cells. All gene information was obtained from genecards.org.

Gene names, t-scores	Description	Key words
APOL1<br>4.02	**Apolipoprotein L1.** This gene encodes a secreted high density lipoprotein which binds to apolipoprotein A-I. The latter is a relatively abundant plasma protein and is the major apoprotein of HDL. It is involved in the formation of most cholesteryl esters in plasma and also promotes efflux of cholesterol from cells. This apolipoprotein L family member may play a role in lipid exchange and transport throughout the body, as well as in reverse cholesterol transport from peripheral cells to the liver.	Transport molecule
C3orf38<br>−4.29/−4.14	**Chromosome 3 open reading frame 38.** May be involved in apoptosis regulation. High expression in testis and bone marrow.	Apoptosis?
GRB10<br>4.28	**Growth factor receptor bound protein 10.** The product of this gene belongs to a small family of adapter proteins that are known to interact with a number of receptor tyrosine kinases and signaling molecules. This gene encodes a growth factor receptor-binding protein that interacts with insulin and insulin-like growth-factor receptors. Over-expression of some isoforms of the encoded protein inhibits tyrosine kinase activity and results in growth suppression.	Signaling
HSPA12A<br>4.84	**Heat shock protein family A member 12A.** Belongs to the HSP70 family. By similarity (HSPA1A): in conjunction with other HSPs, the protein stabilizes existing proteins against aggregation and mediates the folding of newly translated proteins in the cytosol and in organelles. It is also involved in the ubiquitin-proteasome pathway through interaction with the AU-rich element RNA-binding protein 1.	Stress response
KIAA1383/MTR120<br>4.32	**Microtubule regulator 120 kDa.** The protein plays a role in the regulation of cell division, promotes microtubule stability and participates in the organization of the spindle midzone and normal progression of cytokinesis.	Cell cycle, mitosis
LMLN<br>4.22	**Leishmanolysin like peptidase.** This gene encodes a zinc-metallopeptidase. The protein may play a role in cell migration and invasion. Studies of a similar protein in *Drosophila* indicate a potential role in mitotic progression.	Cell migration, cell cycle
NUDT19<br>−4.53	**Nudix hydrolase 19.** By similarity: coenzyme A diphosphatase that mediates the hydrolysis of a wide range of CoA esters, including choloyl-CoA and branched-chain fatty-acyl-CoA esters. At low substrate concentrations, medium and long-chain fatty-acyl-CoA esters are the primary substrates.	Metabolism
P2RX4<br>−4.11	**Purinergic receptor P2X 4.** The product of this gene belongs to the family of purinoceptors for ATP. The receptor functions as a ligand-gated ion channel with high calcium permeability. The main pharmacological distinction between the members of the purinoceptor family is the relative sensitivity to the antagonists suramin and PPADS. The product of this gene has the lowest sensitivity for these antagonists.	Receptor, signaling
PARD6A<br>4.07	**Par-6 family cell polarity regulator α.** This gene is a member of the PAR6 family and encodes a protein with a PSD95/Discs-large/ZO1 domain and a semi-CDC42/Rac interactive binding (CRIB) domain. This cell membrane protein is involved in asymmetrical cell division and cell polarization processes as a member of a multi-protein complex. The protein also has a role in the epithelial-to-mesenchymal (EMT) transition that characterizes the invasive phenotype associated with metastatic carcinomas.	Cell cycle, EMT transition
PPAPDC3/PLPP7	**Phospholipid phosphatase 7 (inactive).** Plays a role as negative regulator of myoblast differentiation, in part through effects	Signaling

4.20	on mTOR signaling. Has no detectable enzymatic activity.	
RPS4Y1 −4.00	**Ribosomal proteinS4, Y-linked 1.** The only ribosomal protein known to be encoded by more than one gene, namely this gene and ribosomal protein S4, X-linked (RPS4X). The two isoforms encoded by these genes are not identical, but functionally equivalent.	Transcription
TMLHE −4.90	**Trimethyllysine hydroxylase ε.** The encoded protein is the first enzyme in the carnitine biosynthesis pathway. Carnitine play an essential role in the transport of activated fatty acids across the inner mitochondrial membrane. The encoded protein converts trimethyllysine into hydroxytrimethyllysine	Cellular transport, metabolism

Table S7. Overview over the mutational landscape of 35 consecutive patients. Patients with more clonogenic cells show more frequently mutations in the spliceosome-cohesin gene groups.

		Colony number		
		**<200 (n=27)**	**≥200 (n=8)**	***p*-value**
Signaling	*Flt3*-ITD	5 (19%)	3 (38%)	0.346
	Flt3-TKD	6 (22%)	0	0.299
	HRAS	1 (4%)	0	1.000
	JAK2	1 (4%)	0	1.000
	KIT	1 (4%)	0	1.000
	KRAS	3 (11%)	1 (13%)	1.000
	NOTCH1	1 (4%)	0	1.000
	NRAS	5 (19%)	2 (25%)	0.648
	PTPN11	2 (7%)	1 (13%)	0.553
	Total group	25 mutations	7 mutations	1.000
Tumor suppressors	*CDKN2A*	1 (4%)	0	1.000
	IKZF1	4 (15%)	0	0.553
	PHF6	1 (4%)	0	1.000
	TP53	1 (4%)	1 (13%)	0.410
	WT1	2 (7%)	0	1.000
	Total group	9 mutations	1 mutation	0.458
Epigenetic modifiers	*ASXL1*	2 (7%)	3 (38%)	0.067
	DNMT3A	8 (30%)	3 (38)	0.685
	EZH2	2 (7%)	1 (13%)	0.553
	IDH1	2 (7%)	0	1.000
	IDH2	4 (15%)	0	0.553
	KMT2A/MLL	1 (4%)	0	1.000
	TET2	4 (15%)	3 (38%)	0.312
	Total group	23 mutations	10 mutations	0.272
Myeloid TFs	*CEBPA*	4 (15%)	1 (13%)	1.000
	GATA2	2 (7%)	1 (13%)	0.553
	RUNX1	5 (19%)	2 (25%)	0.648
	Total group	11 mutations	4 mutations	0.743
Spliceosome/ transcription repressors	*BCOR*	0	1 (13%)	0.229
	BCORL1	0	1 (13%)	0.229
	SF3B1	1 (4%)	1 (13%)	0.410
	SRSF2	3 (11%)	1 (13%)	1.000
	Total group	4 mutations	4 mutations	0.080
Cohesin	*RAD21*	0	1 (13%)	0.229
	SMC1A	1 (4%)	0	1.000
	STAG2	1 (4%)	2 (25%)	0.124
	Total group	2 mutations	3 mutations	0.077
Others	*CSF3R*	1 (4%)	0	1.000
	NPM1-ins	4 (15%)	3 (38%)	0.312
	SETBP1	1 (4%)	0	1.000
	Total number	66	27	0.157
	Per patient	2.4	3.4	

No mutation in *CUX1*, *KDM6A* and *ZRSR2* for any patient.

Table S8. Single genes identified in the comparison of global gene expression profiles for patients with and without signs of morphological differentiation. A total of 58 annotated genes were identified, thereof 35 with a known function. Genes with a positive t-score are overexpressed in the group that shows an altered phenotype. All gene information was obtained from genecards.org. The proteins with acknowledged impact in AML are highlighted. PubMed Identifiers (PMIDs) are provided.

Gene names, t-scores	Description	Key words
AKR1D1 −4.08	**Aldo-keto reductase family 1 member D1.** The enzyme encoded by this gene is responsible for the catalysis of the 5β-reduction of bile acid intermediates and steroid hormones carrying a δ(4)-3-one structure. Deficiency of this enzyme may contribute to hepatic dysfunction.	Steroid binding, metabolism
AMZ2 −5.33	**Archaelysin family metallopeptidase 2.** The protein encoded by this gene is a zinc metalloprotease that displays some activity against angiotensin-3. The encoded protein is inhibited by the aminopeptidase inhibitor amastatin, as well as by the general inhibitors o-phenanthroline and batimastat. Defects in this gene may be associated with lung tumorigenesis.	Metabolism
BRF1 −4.25	**RNA polymerase III transcription initiation factor 90 kDa subunit.** This gene encodes one of the three subunits of the RNA polymerase III transcription factor complex. This complex plays a central role in transcription initiation by RNA polymerase III on genes encoding tRNA, 5S rRNA, and other small structural RNAs. The gene product belongs to the TF2B family.	Transcription
BVES −4.11	**Blood vessel epicardial substance.** This gene encodes a member of the POP family of proteins containing three putative transmembrane domains. This gene is expressed in cardiac and skeletal muscle and may play an important role in development of these tissues. The mouse ortholog may be involved in the regeneration of adult skeletal muscle and may act as a cell adhesion molecule in coronary vasculogenesis.	Tissue development, cell adhesion
C10orf59/RNLS 4.04	**Renalase.** Renalase is a flavin adenine dinucleotide-dependent amine oxidase that is secreted into the blood from the kidney, where it modulates cardiac function and systemic blood pressure.	Metabolism, heart rate
CANX 4.72/4.20	**Calnexin.** This gene encodes a member of the calnexin family of molecular chaperones. The encoded protein is a calcium-binding, endoplasmic reticulum (ER)-associated protein that interacts transiently with newly synthesized N-linked glycoproteins, facilitating protein folding and assembly. It may also play a central role in the quality control of protein folding by retaining incorrectly folded protein subunits within the ER for degradation	Chaperone
CARD11 −5.19	**Caspase recruitment domain family member 11.** Involved in the costimulatory signal essential for T-cell receptor (TCR)-mediated T-cell activation. Its binding to dipeptidyl peptidase 4 induces T-cell proliferation and NFκB activation in a T-cell receptor/CD3-dependent manner. Activates NFκB via Bcl10 and IKK. Stimulates the phosphorylation of Bcl10. Also activates the TORC1 signaling pathway.	Signaling, T-cell proliferation
CBLB −4.11	**Cbl proto-oncogene B.** This gene encodes an E3 ubiquitin-protein ligase which promotes proteosome-mediated protein degradation by transferring ubiquitin from an E2 ubiquitin-conjugating enzyme to a substrate. The encoded protein is involved in the regulation of immune response by limiting T-cell receptor, B-cell receptor, and high affinity immunoglobulin ε receptor activation. Studies in mouse suggest that this gene is involved in antifungal host defense and that its inhibition leads to increased fungal killing. Manipulation of this gene may be beneficial in implementing immunotherapies for a variety of conditions, including cancer, autoimmune diseases, allergies, and infections.	Ubiquitination, immune response
CHP 4.55	**Calcineurin like EF-hand protein 1.** This gene encodes a phosphoprotein that binds to the Na^+/H^+ exchanger NHE1. This protein serves as an essential cofactor which supports the physiological activity of NHE family members and may play a role in the	Metabolism, trafficking,

	mitogenic regulation of NHE1. The protein shares similarity with calcineurin B and calmodulin and it is also known to be an endogenous inhibitor of calcineurin activity. It is further involved in different processes such as regulation of vesicular trafficking, and gene transcription. Involved in the constitutive exocytic membrane traffic. Mediates the association between microtubules and membrane-bound organelles of the endoplasmic reticulum and Golgi apparatus and is also required for the targeting and fusion of transcytotic vesicles with the plasma membrane.	transcription
CSMD1 −4.17	**CUB and Sushi multiple domains 1.** Potential suppressor of squamous cell carcinomas.	
CTAGE5/MIA2 4.20	**MIA SH3 domain ER export factor 2.** This gene encodes a receptor in the ER, which plays a role in the export of large pre-chylomicrons and pre-very low density lipoproteins. Three major classes of transcripts are generated from this gene—melanoma inhibitory activity 2-specific transcripts, cTAGE family member 5-specific transcripts and transcripts that include exons from both these transcript species. Associated with lymphoma.	Metabolism
D2HGDH −4.71	**D-2-hydroxygluturate dehydrogenase.** A mitochondrial enzyme belonging to the FAD-binding oxidoreductase/transferase type 4 family. This enzyme, which is most active in liver and kidney but also active in heart and brain, converts D-2-hydroxyglutarate to 2-ketoglutarate.	Metabolism
DMTF1 −5.93/-5.55	**Cyclin D binding myb like transcription factor 1.** This gene encodes a transcription factor that contains a cyclin D-binding domain, three central myb-like repeats, and two flanking acidic transactivation domains at the N- and C-termini. The encoded protein is induced by the oncogenic Ras signaling pathway and functions as a tumor suppressor by activating the transcription of ARF and thus the ARF-p53 pathway to arrest cell growth or induce apoptosis. It also activates the transcription of aminopeptidase N and may play a role in hematopoietic cell differentiation. The transcriptional activity of this protein is regulated by binding of D-cyclins. Suppressor of malignant transformation in AML (PMID: 10095122). Plays potentially a role in hematopoietic cell differentiation (PMID: 12917399).	Transcription, tumor suppressor, cell differentiation
DUSP15 −5.02	**Dual specificity phosphotase 15.** The protein encoded by this gene has both protein-tyrosine phosphatase activity and serine/threonine-specific phosphatase activity, and therefore is known as a dual specificity phosphatase. This protein may function in the differentiation of oligodendrocytes.	Metabolism, differentiation
E2F5 −4.16	**E2F transcription factor 5.** The protein encoded by this gene is a member of the E2F family of transcription factors. The E2F family plays a crucial role in the control of cell cycle and action of tumor suppressor proteins and is also a target of the transforming proteins of small DNA tumor viruses. The E2F proteins contain several evolutionarily conserved domains that are present in most members of the family. These domains include a DNA binding domain, a dimerization domain, which determines interaction with the differentiation regulated transcription factor proteins, a transactivation domain enriched in acidic amino acids, and a tumor suppressor protein association domain which is embedded within the transactivation domain. This protein is differentially phosphorylated and is expressed in a wide variety of human tissues.	Transcription, cell cycle
FBXO44 −4.19	**F-box protein 44.** This gene encodes a member of the F-box protein family, which is characterized by an approximately 40 amino acid motif, the F-box. The F-box proteins constitute one of the four subunits of the ubiquitin protein ligase complex called SCFs (SKP1-cullin-F-box), which function in phosphorylation-dependent ubiquitination.	Ubiquitination
FOXR1 −4.07	**Forkhead box R1.** This gene encodes a member of the forkhead box (FOX) family of transcription factors. FOX family members are monomeric, helix-turn-helix proteins with a core DNA-binding domain of approximately 110 aa. Many FOX transcription factors play roles in determining cell fates during early development. This forkhead box protein lacks the C-terminal basic region found in many other FOX family members.	Transcription

FUZ<br>−4.52	**Fuzzy planar cell polarity protein.** This gene encodes a planar cell polarity protein that is involved in ciliogenesis and directional cell movement. Knockout studies in mice exhibit neural tube defects and defective cilia, and mutations in this gene are associated with neural tube defects in humans.	Signaling, cell movement
GNRH1<br>−4.49	**Gonadotropin releasing hormone 1.** Stimulates the secretion of gonadotropins; it stimulates the secretion of both luteinizing and follicle-stimulating hormones.	Signaling, hormone activity
H2AFY<br>4.15	**H2A histone family member Y.** Histones are basic nuclear proteins that are responsible for the nucleosome structure of the chromosomal fiber in eukaryotes. This gene encodes a replication-independent histone that is a member of the histone H2A family. It replaces conventional H2A histones in a subset of nucleosomes where it represses transcription and participates in stable X chromosome inactivation.	Transcription inhibitor
HEXA<br>4.25	**Hexosaminidase subunit α.** This gene encodes a member of the glycosyl hydrolase 20 family of proteins. The encoded pre-proprotein is proteolytically processed to generate the α subunit of the lysosomal enzyme β-hexosaminidase. This enzyme, together with the cofactor GM2 activator protein, catalyzes the degradation of the ganglioside GM2, and other molecules containing terminal N-acetyl hexosamines.	Metabolism
KRT6A<br>4.10	**Keratin 6A.** The protein encoded by this gene is a member of the keratin gene family. The type II cytokeratins consist of basic or neutral proteins, which are arranged in pairs of heterotypic keratin chains co-expressed during differentiation of simple and stratified epithelial tissues. As many as six of this type II cytokeratin have been identified; the multiplicity of the genes is attributed to successive gene duplication events. The genes are expressed with family members KRT16 and/or KRT17 in the filiform papillae of the tongue, the stratified epithelial lining of oral mucosa and esophagus, the outer root sheath of hair follicles, and the glandular epithelia. This KRT6 gene in particular encodes the most abundant isoform. In addition, peptides from the C-terminal region of the protein have antimicrobial activity against bacterial pathogens.	Wound healing, antimicrobial effect
MAPK15<br>−4.15	**Mitogen-activated protein kinase 15.** Putative MAP kinase. Phosphorylates myelin basic protein *in vitro*.	Metabolism, signaling
MC1R<br>−4.38	**Melanocortin 1 receptor.** This intron-less gene encodes the receptor protein for melanocyte-stimulating hormone (MSH). The encoded protein, a seven pass transmembrane G protein coupled receptor, controls melanogenesis. This receptor is a major determining factor in sun sensitivity and is a genetic risk factor for melanoma and non-melanoma skin cancer.	Signaling
MRC1<br>−5.11	**Mannose receptor C type 1.** The recognition of complex carbohydrate structures on glycoproteins is an important part of several biological processes, including cell-cell recognition, serum glycoprotein turnover, and neutralization of pathogens. The protein encoded by this gene is a type I membrane receptor that mediates the endocytosis of glycoproteins by macrophages. The protein has been shown to bind high-mannose structures on the surface of potentially pathogenic viruses, bacteria, and fungi so that they can be neutralized by phagocytic engulfment.	Immune response
NXNL1<br>−4.76	**Nucleoredoxin-like 1.** Retinitis pigmentosa (RP) is a disease that leads to blindness by degeneration of cone photoreceptors. Rods produce factors required for cone viability. The protein encoded by this gene is one of those factors and is similar to a truncated form of thioredoxin.	Vision
POLR2B<br>4.02	**RNA polymerase II subunit B.** This gene encodes the second largest subunit of RNA polymerase II (Pol II), a DNA-dependent RNA polymerase that catalyzes the transcription of DNA into precursors of mRNA, snRNA and microRNA. This subunit and the largest subunit form opposite sides of the center cleft of Pol II. Deletion of the flap loop region of this subunit results in a decrease in the rate of transcriptional elongation.	Transcription
PPM1B	**Protein phosphatase, Mg^{2+}/Mn^{2+} dependent 1B.** The protein encoded by this gene is a member of the PP2C family of Ser/Thr	Cell cycle

−4.11	protein phosphatases. PP2C family members are known to be negative regulators of cell stress response pathways. This phosphatase has been shown to dephosphorylate cyclin-dependent kinases (CDKs), and thus may be involved in cell cycle control. Overexpression of this phosphatase is reported to cause cell-growth arrest or cell death.	
RHOD −4.21	**Ras homolog family member 2.** Ras homolog, or Rho, proteins interact with protein kinases and may serve as targets for activated GTPase. They play a critical role in muscle differentiation. The protein encoded by this gene binds GTP and is a member of the small GTPase superfamily. It is involved in endosome dynamics and reorganization of the actin cytoskeleton, and it may coordinate membrane transport with the function of the cytoskeleton.	Trafficking, cytoskeleton
SCNN1D −4.13	**Sodium channel epithelial 1δ subunit.** The sodium permeable non-voltage-sensitive ion channel is inhibited by the diuretic amiloride. It mediates the electrodiffusion of the luminal sodium (and water, which follows osmotically) through the apical membrane of epithelial cells. Controls the reabsorption of sodium in kidney, colon, lung and sweat glands. Also plays a role in taste perception.	Trafficking
SMAD2 4.08	**SMAD family member 2.** SMAD proteins are signal transducers and transcriptional modulators that mediate multiple signaling pathways. This protein mediates the signal of TGF-β, and thus regulates multiple cellular processes, such as cell proliferation, apoptosis, and differentiation. This protein is recruited to the TGF-β receptors through its interaction with the SMAD anchor for receptor activation protein. In response to TGF-β signal, this protein is phosphorylated by the receptors, which induces the association with the family member SMAD4. The association with SMAD4 is important for the translocation of this protein into the nucleus, where it binds to target promoters and forms a transcription repressor complex with other cofactors. Promotes leukemogenesis (PMID: 23725749).	Signaling, transcription
SPAG9 −4.98	**Sperm associated antigen 9.** This gene encodes a member of the cancer testis antigen gene family. The encoded protein functions as a scaffold protein that structurally organizes MAP kinases and mediates c-Jun-terminal kinase signaling. This protein also binds to kinesin-1 and may be involved in microtubule-based membrane transport. This protein may play a role in tumor growth and development.	Signaling
SPINK6 −4.25	**Serine peptidase inhibitor Kazal type 6.** The protein encoded by this gene is a Kazal-type serine protease inhibitor that acts on kallikrein-related peptidases in the skin.	Metabolism
TMTC4 −4.12	**Transmembrane and tetratricopeptide repeat containing 4.** This gene encodes a transmembrane protein that belongs to family of proteins containing an N-terminal transmembrane domain and a C-terminal tetratricopeptide repeat (TPR) domain. TPR domains mediate protein-protein interactions in various cellular processes, such as synaptic vesicle fusion, protein folding, and protein translocation.	Protein folding
TOR1AIP1 4.23	**Torsin 1A interacting protein 1.** This gene encodes a type 2 integral membrane protein that binds A- and B-type lamins. The encoded protein localizes to the inner nuclear membrane and may be involved in maintaining the attachment of the nuclear membrane to the nuclear lamina during cell division.	Cytosekeleton

Table S9. Classification of the genes that were found to be differentially expressed for patient populations with change in their phenotype.

Classification based on protein function	Gene expression in AML population with phenotype change	
	**Increased**	**Decreased**
Cell cycle		*E2F5; PPM1B*
Transcription, RNA binding	*CHP; POLR2B; SMAD2*	*BRF1; DMTF1; E2F5; FOXR1*
Transcription inhibition	*H2AFY*	
Chaperone	*CANX*	*TMTC4*
Cell adhesion, cytoskeletal/adhesion proteins	*KRT6A; TOR1AIP1*	*BVES; RHOD*
Trafficking	*CHP*	*RHOD; SCNN1D*
Tumor suppression		*DMTF1*
Cell differentiation		*DMTF1; DUSP15*
Signaling	*SMAD2*	*CARD11; FUZ; GNRH1; MC1R; MAPK15; SPA9*
Metabolism, mitochondria	*C10orf59/RNLS; CHP; CTAGE5/MIA2; HEXA*	*AKR1D1; AMZ2; D2HGDH; DUSP15; MAPK15; SPINK6*
Antimicrobial response, immune system	*KRT6A*	*CBLB; MRC1*
Ubiquitination		*CBLB; FBOX44*
Vision		*NXNL1*
Unknown, pseudogenes, hypothetical genes	*LOC651476; LOC728059; LOC730376; MFSD1*	*C20orf195/FNDC11; CSMD1; DBNDD1; FLJ45244; LOC169834; LOC440348; LOC440353; LOC440366; LOC613037/NPIPB13; LOC727735; LOC727913; LOC728888; LOC731102; LOC100008589; LOC100132247; NPIP; SNORA17; TBC1D3G*

Table S10. Single genes identified in the comparison of global gene expression profiles for patients with and without signs erythroid colonies. A total of 36 annotated genes were identified, thereof 23 with a known function. Genes with a positive t-score are upregulated in the group that shows colonies of erythroid origin. All gene information was obtained from genecards.org. The proteins with acknowledged impact in AML are highlighted and PubMed Identifiers (PMIDs) are provided.

Gene names, t-scores	Description	Key words
BZW1<br>−4.22	**Basic leucine zipper and W2 domains 1.** Enhances histone H4 gene transcription but does not seem to bind DNA directly.	Transcription
C2orf71/PCARE<br>4.14	**Photoreceptor cilium actin regulator.** The protein encoded by this gene is highly expressed in photoreceptors and may associate with the primary cilium of the outer segment. The encoded protein appears to undergo post-translational lipid modification. Nonsense and missense variants of this gene appear to cause a recessive form of retinitis pigmentosa.	Vision
C21orf66/PAXBP1<br>4.20	**PAX3 and PAX7 binding protein 1.** This gene encodes a protein that may bind to GC-rich DNA sequences, which suggests its involvement in the regulation of transcription.	Transcription
CASC2<br>4.54	**Cancer susceptility 2.** CASC2 is an RNA gene. It may act as a potential tumor suppressor.	Tumor suppressor
CASD1<br>4.28	**CAS domain containing 1.** O-acetyltransferase that catalyses 9-O-acetylation of sialic acids.	Metabolism
CEP78<br>4.01	**Centrosomal protein 78.** This gene encodes a centrosomal protein that is both required for the regulation of centrosome-related events during the cell cycle, and required for ciliogenesis. It interacts with the N-terminal catalytic domain of polo-like kinase 4 (PLK4) and colocalizes with PLK4 to the distal end of the centriole. Naturally occurring mutations in this gene cause defects in primary cilia that result in retinal degeneration and sensorineural hearing loss which are associated with cone-rod degeneration disease as well as Usher syndrome. Low expression of this gene is associated with poor prognosis of colorectal cancer patients.	Cell cycle
CLPP<br>4.02	**Caseinolytic mitochondrial matrix peptidase proteolytic subunit.** The protein encoded by this gene belongs to the peptidase family S14 and hydrolyzes proteins into small peptides in the presence of ATP and magnesium. The protein is transported into mitochondrial matrix and is associated with the inner mitochondrial membrane.<br>Marker of mitochondrial stress and essential for AML cell viability (PMIDs: 26058072, 26058080).	Metabolism
DCX<br>−4.62	**Doublecortin.** This gene encodes a member of the doublecortin family. The protein encoded by this gene is a cytoplasmic protein and contains two doublecortin domains, which bind microtubules. The encoded protein appears to direct neuronal migration by regulating the organization and stability of microtubules. In addition, the encoded protein interacts with LIS1, the regulatory gamma subunit of platelet activating factor acetylhydrolase, and this interaction is important to proper microtubule function in the developing cortex.	Cortex development, cytoskeletal signaling
ENTPD8<br>4.56	**Ectonucleoside triphosphate diphosphohydrolase 8.** Ectonucleoside NTPDases catalyze the hydrolysis of β- and γ-phosphate residues of nucleotides, playing a central role in concentration of extracellular nucleotides. Has activity toward ATP, ADP, UTP and UDP, but not toward AMP.	Metabolism
FGF12<br>4.13	**Fibroblast growth factor 12.** The protein encoded by this gene is a member of the fibroblast growth factor (FGF) family. FGF family members possess broad mitogenic and cell survival activities, and are involved in a variety of biological processes, including embryonic development, cell growth, morphogenesis, tissue repair, tumor growth, and invasion. This growth factor lacks the N-terminal signal sequence present in most of the FGF family members, but it contains clusters of basic residues that have been	Signaling molecule

	demonstrated to act as a nuclear localization signal. The specific function of this gene has not yet been determined.	
GNAT3<br>4.34	**Guanine nucleotide binding protein α transducing 3.** The protein plays a prominent role in bitter, sweet and umami taste transduction. Transduction by this α subunit involves coupling of specific cell-surface receptors with a cGMP-phosphodiesterase. Activation of phosphodiesterase lowers intracellular levels of cAMP and cGMP, which may open a cyclic nucleotide-suppressible cation channel leading to influx of calcium, ultimately leading to release of neurotransmitter. GNAT3 may also modulate the gut capacity to absorb sugars, with implications in malabsorption syndromes and diet-related disorders including diabetes and obesity.	Signal transducer, metabolic syndrome
HP1BP3<br>4.20	**Heterochromatin protein 1 binding protein 3.** Component of heterochromatin that maintains heterochromatin integrity during G_1/S progression and regulates the duration of G_1 phase to critically influence cell proliferative capacity. Mediates chromatin condensation during hypoxia, leading to increased tumor cell viability, radio-resistance, chemo-resistance and self-renewal.	Cell cycle
LHX3<br>4.44	**LIM homeobox 3.** This gene encodes a member of a large family of proteins, which carry the LIM domain, a unique cysteine-rich zinc-binding domain. The encoded protein is a transcription factor that is required for pituitary development and motor neuron specification. Mutations in this gene cause combined pituitary hormone deficiency 3.	Transcription
OR52D1<br>−4.04	**Olfactory receptor 52D1.** Olfactory receptors interact with odorant molecules in the nose, to initiate a neuronal response that triggers the perception of a smell. The olfactory receptor proteins are members of a large family of G-protein-coupled receptors (GPCR) arising from single coding-exon genes.	Receptor signaling
PHB<br>4.04	**Prohibitin.** The protein inhibits DNA synthesis. It has a role in regulating proliferation and is further proposed to play a role in human cellular senescence and tumor suppression.	Cell cycle inhibition
PPM1B<br>4.06	**Protein phosphatase Mg^{2+}/Mn^{2+} dependent 1.** The protein encoded by this gene is a member of the PP2C family of Ser/Thr protein phosphatases. PP2C family members are known to be negative regulators of cell stress response pathways. This phosphatase has been shown to dephosphorylate cyclin-dependent kinases (CDKs), and thus may be involved in cell cycle control. Overexpression of this phosphatase is reported to cause cell-growth arrest or cell death.	Cell cycle control
PRDM8<br>4.02	**PR/SET domain 8.** This gene encodes a protein that belongs to a conserved family of histone methyltransferases that predominantly act as negative regulators of transcription. The encoded protein contains an N-terminal Su(var)3-9, Enhancer-of-zeste, and Trithorax (SET) domain and a double zinc-finger domain. Knockout of this gene in mouse results in mistargeting by neurons of the dorsal telencephalon, abnormal itch-like behavior, and impaired differentiation of rod bipolar cells. In humans, the protein has been shown to interact with the phosphatase laforin and the ubiquitin ligase malin, which regulate glycogen construction in the cytoplasm.	Transcription regulation
RALBP1<br>−4.09	**RalA binding protein 1.** RALBP1 plays a role in receptor-mediated endocytosis and is a downstream effector of the small GTP-binding protein RAL. Mediates ATP-dependent transport of S-(2,4-dinitrophenyl)-glutathione (DNP-SG) and doxorubicin (DOX) and is the major ATP-dependent transporter of glutathione conjugates of electrophiles (GS-E) and DOX in erythrocytes. Can catalyze transport of glutathione conjugates and xenobiotics, and may contribute to the multidrug resistance phenomenon. Serves as a scaffold protein that brings together proteins forming an endocytotic complex during interphase and also with CDK1 to switch off endocytosis.	Transport protein, drug resistance
RPL28<br>−6.78	**Ribosomal protein L28.** This gene encodes a ribosomal protein that is a component of the 60S subunit. The protein belongs to the L28E family of ribosomal proteins. It is located in the cytoplasm.	Protein synthesis
RTN3<br>4.20	**Reticulon 3.** This gene belongs to the reticulon family of highly conserved genes that are preferentially expressed in neuroendocrine tissues. This family of proteins interact with, and modulate the activity of β-amyloid converting enzyme 1 (BACE1), and the production of amyloid-β. Inhibits BACE1 activity and amyloid precursor protein processing. May be involved in membrane trafficking in the early secretory pathway. May induce caspase-8 cascade and apoptosis. May favor BCL2 translocation to the mitochondria upon endoplasmic reticulum stress. In case of enteroviruses infection, RTN3 may be involved in the viral replication or pathogenesis.	Trafficking, apoptosis

	Induces the formation of endoplasmic reticulum tubules.	
SLC7A6OS<br>4.67	**Solute carrier family 7 member opposite strand.** Directs RNA polymerase II nuclear import.	Membrane trafficking
UHRF1<br>4.13	**Ubiquitin-like with PHD and RING finger domains 1.** This gene encodes a member of a subfamily of RING-finger type E3 ubiquitin ligases. The protein binds to specific DNA sequences, and recruits a histone deacetylase to regulate gene expression. Its expression peaks at late G_1 phase and continues during G_2 and M phases of the cell cycle. It plays a major role in the G_1/S transition by regulating topoisomerase IIα and retinoblastoma gene expression, and functions in the p53-dependent DNA damage checkpoint. It is regarded as a hub protein for the integration of epigenetic information. This gene is up-regulated in various cancers, and it is therefore considered to be a therapeutic target.<br>Decreased DNA methylation and low cytogenetic risk (PMID: 25996682).	Transcription, cell cycle control
ZNF296<br>−4.08	**Zinc finger protein 296.** May be a transcriptional corepressor with KLF4.	Transcription

Table S11. Classification of the genes that were found to be differentially expressed for patient populations, which showed colonies of erythroid origin.

**Classification based on protein function**	**Gene expression in AML population with erythroid colony formation**	
	**Increased**	**Decreased**
Cell cycle, mitosis	*CEP78; HP1BP3*	
Transcription	*C21orf66/PAXBP1; LHX3; PRDM8; UHRF1*	*BZW1; ZNF296?*
Translation		*RPL28*
Tumor suppressor, cell cycle arrest	*CASC2; PHB; PPM1B*	
Metabolism	*CASD1; CLPP; ENTPD8; GNAT3; UHRF1*	
Membrane trafficking	*RTN3; SLC7A6OS*	
Signaling, transport	*FGF12; GNAT3*	*DCX; OR52D1; RALBP1*
Vision	*C2orf71/PCARE; CEP78*	
Unknown, pseudogenes, hypothetical genes	*C7orf38/FAM200A; FAM187B; FAM90A20; LOC642650; LOC646429; LOC650029; LOC652703; LOC728951; LOC729057; LOC100132550*	*KIAA1539/FAM214B; LOC44316; LOC729683*

Table S12. Comparison of the proteomic profile at the time of diagnosis for primary human AML cells derived from patients with many and few/no colony-forming cells. All protein information was obtained from genecards.org. Proteins that have been studied in AML are highlighted and PubMed Identifiers (PMIDs) are provided.

**Protein names, *p*-values**	**Description, increased in cultures ≥200 colonies**	**Keywords**
C19orf43/TRIR 0.016	**Telomerase RNA component interacting RNase.** Exoribonuclease that is part of the telomerase RNA 3 end processing complex and which has the ability to all four unpaired RNA nucleotides from 5` or 3` with higher efficiency for purine bases.	Telomerase
CLIC4 0.019	**Chloride intracellular channel 4.** Chloride channels are a diverse group of proteins that regulate fundamental cellular processes including stabilization of cell membrane potential, transepithelial transport, maintenance of intracellular pH, and regulation of cell volume.	Hepatic ABC transporter, ion channel
CPD 0.006	**Carboxypeptidase D.** The metallocarboxypeptidase family of enzymes is divided into 2 subfamilies based on sequence similarities: the pancreatic carboxypeptidase-like and the regulatory B-type carboxypeptidase subfamilies. Carboxypeptidase D has been identified as a member of the latter subfamily.	Transporter, carboxypeptidase
CRIP1 0.035	**Cysteine-rich protein 1.** CRIP1 belongs to the LIM/double zinc finger protein family (transcription factors), and may be involved in intestinal zinc transport.	Transporter
GPALPP1 0.036	**GPALPP motifs containing 1.** Is also expressed in normal bone marrow.	?
HEXB 0.026	**Hexosaminidase subunit β.** Hexosaminidase B is the β subunit of the lysosomal enzyme β-hexosaminidase that, together with the cofactor GM2 activator protein, catalyzes the degradation of the ganglioside GM2, and other molecules containing terminal N-acetyl hexosamines.	Hydrolase, lysosome
MED1 0.024	**Mediator complex subunit 1.** The activation of gene transcription is a multistep process that is triggered by factors that recognize transcriptional enhancer sites in DNA. These factors work with co-activators to direct transcriptional initiation by the RNA polymerase II apparatus. This protein is a subunit of the CRSP (cofactor required for SP1 activation) complex, which, along with TFIID, is required for efficient activation by SP1. It is also a component of other multisubunit complexes e.g. thyroid hormone receptor-(TR-) associated proteins, which interact with TR and facilitate TR function on DNA templates in conjunction with initiation factors and cofactors. It also regulates p53-dependent apoptosis and it is essential for adipogenesis. The protein is known to have the ability to self-oligomerize.	Transcription, apoptosis
MOB4 0.020	**MOB family member 4.** Studies of the mouse counterpart suggest that the expression of this gene may be regulated during oocyte maturation and preimplantation following zygotic gene activation. Diseases associated with MOB4 include myeloproliferative syndrome.	Kinase binding
MPDU1 0.047	**Mannose-P-dolichol utilization defect 1.** An endoplasmic reticulum membrane protein that is required for utilization of the mannose donor mannose-P-dolichol in the synthesis of lipid-linked oligosaccharides and glycosylphosphatidylinositols.	Metabolism, endoplasmatic reticulum
POLR3C 0.047	**RNA polymerase III subunit C.** Diseases associated with POLR3C include chickenpox. Among its related pathways are RNA polymerase III transcription initiation and pyrimidine metabolism. High expression in normal bone marrow.	Transcription

RALGAPA2 0.031	**Ral GTPase activating protein catalytic α subunit 2.** Among its related pathways are vesicle-mediated transport and translocation of GLUT4 to the plasma membrane. High expression in normal bone marrow.	Transporter, signaling
RBMXL1 0.043	**RBMX like 1.** This gene represents a retro-gene of RNA binding motif protein, X-linked, which is located on chromosome X. The locus is likely to be functional, possibly during male meiosis when X chromosomal genes are silenced or during haploid stages of spermatogenesis.	pre-mRNA splicing
RMDN3 0.025	**Regulator of microtubule dynamics 3.** Involved in cellular calcium homeostasis regulation. May participate in differentiation and apoptosis of keratinocytes. Overexpression induces apoptosis. Expression in normal bone marrow.	Calcium homeostasis, apoptosis
TMEM173 0.050	**Transmembrane protein 173.** A five transmembrane protein that functions as a major regulator of the innate immune response to viral and bacterial infections. The protein is a pattern recognition receptor that detects cytosolic nucleic acids and transmits signals that activate type I interferon responses. It has also been shown to play a role in apoptotic signaling by associating with type II major histocompatibility complex.	Immune response, apoptotic signaling
TXNL4A 0.009	**Thioredoxin like A4.** The protein is a member of the U5 small ribonucleoprotein particle (snRNP), and is involved in pre-mRNA splicing.	pre-mRNA splicing
VIM (PMID: 29925392) 0.036	**Vimentin.** This type III intermediate filament protein, along with microtubules and actin microfilaments, make up the cytoskeleton. The protein is responsible for maintaining cell shape and integrity of the cytoplasm, and stabilizing cytoskeletal interactions. It is involved in cholesterol transport and functions as an organizer of a number of other critical proteins involved in cell attachment, migration, and signaling.	(Cytoskeletal) signaling, adhesion
ZNF830 0.008	**Zinc finger protein 830.** Among its related pathways are transcription-coupled nucleotide excision repair and DNA double-strand break repair.	pre-mRNA splicing?, Transcription

**Protein names, *p*-values**	**Description, decreased in cultures ≥200 colonies**	**Keywords**
AAMP 0.048	**Angio-associated migratory cell protein.** A member of the immunoglobulin superfamily. The protein is associated with angiogenesis, with potential roles in endothelial tube formation and the migration of endothelial cells. It can bind to heparin and may mediate heparin-sensitive cell adhesion.	Angiogenesis, migration, adhesion
ABCF1 0.005	**ATP-binding cassette subfamily F, member 1.** The protein is a member of the superfamily of ATP-binding cassette (ABC) transporters. ABC proteins transport various molecules across extra- and intra-cellular membranes. This protein is a member of the GCN20 subfamily. Unlike other members of the superfamily, it lacks the transmembrane domains, which are characteristic of most ABC transporters. This protein may be regulated by tumor necrosis factor α and play a role in enhancement of protein synthesis and the inflammation process.	Transporter, protein synthesis, inflammation?
CAMK2D 0.027	**Calcium/calmodulin dependent protein kinase IIδ.** A member of the serine/threonine protein kinase family and to the Ca^{2+}/calmodulin-dependent protein kinase subfamily. Calcium signaling is crucial for several aspects of plasticity at glutamatergic synapses.	Kinase
CAP1 0.025	**Cyclase associated actin cytoskeleton regulatory protein 1.** The protein is related to the *S. cerevisiae* CAP protein, which is involved in the cyclic AMP pathway. The human protein is able to interact with other molecules of the same protein, as well as with CAP2 and actin.	Cytoskeletal signaling, binding of actin
CLCN3 0.033	**Chloride voltage-gated channel 3.** This is a member of the voltage-gated chloride channel (ClC) family. The protein is present in all cell types and localized in plasma membranes and in intracellular vesicles. The ClC domain catalyzes the selective flow of Cl^-	Hepatic ABC transporter (see

	ions across cell membranes, and the CBS domain may have a regulatory function. The protein activity is regulated by CAMK2 (see above) in glioma cells.	above), ion channel
DGKZ<br>0.022	**Diaglycerol kinase ζ.** The protein belongs to the eukaryotic diacylglycerol kinase family. It may attenuate protein kinase C activity by regulating diacylglycerol levels in intracellular signaling cascade and signal transduction.	Kinase, intrecellular signaling
EIF2B2<br>0.004	**Eukaryotic translation initiation factor 2B subunit β.** EIF2B is involved in protein synthesis and exchanges GDP and GTP for its activation and deactivation.	Protein synthesis
EPM2AIP1<br>0.026	**EPMA2 interacting protein 1.** The protein binds to laforin, but its function is not known.	?
FARSB<br>0.026	**Phenylalanyl-tRNA synthetase subunit β.** This highly conserved enzyme comprises the regulatory β subunits that form a tetramer with two catalytic *α* subunits. In the presence of ATP, this tetramer is responsible for attaching L-Phe to the terminal adenosine of the appropriate tRNA.	RNA metabolism
MAP7D1<br>0.024	**MAP7 domain containing 1.** Expressed in normal bone marrow.	Structural molecule?
MRC2<br>0.039	**Mannose receptor C type 2.** This is a member of the mannose receptor family of proteins that contain a fibronectin type II domain and multiple C-type lectin-like domains. The protein plays a role in extracellular matrix remodeling by mediating the internalization and lysosomal degradation of collagen ligands. Expression of the gene may play a role in the tumorigenesis and metastasis of several malignancies.	Receptor signaling, lysosome
MRPL3<br>0.048	**Mitochondrial ribosomal protein L3.** Mammalian mitochondrial ribosomal proteins are encoded by nuclear genes and help in protein synthesis within the mitochondrion. Mitochondrial ribosomes (mitoribosomes) consist of a small 28S subunit and a large 39S subunit. This 39S subunit protein belongs to the L3P ribosomal protein family.	Mitochondrial ribosome
MYOF<br>0.038	**Myoferlin. A** type II membrane protein that is structurally similar to dysferlin. It is a member of the ferlin family and associates with both plasma and nuclear membranes. The protein contains C2 domains that play a role in calcium-mediated membrane fusion events, suggesting that it may be involved in membrane regeneration and repair.	Membrane binding
RBP4<br>0.037	**Retinol binding protein 4.** This protein belongs to the lipocalin family and is the specific carrier for retinol (vitamin A alcohol) in the blood. It delivers retinol from the liver stores to the peripheral tissues. In plasma, the RBP-retinol complex interacts with transthyretin, which prevents its loss by filtration through the kidney glomeruli.	Retinol transport, Vitamin A
SMG1 (PMID: 25257528)<br>0.046	**Nonsense mediated mRNA decay associated PI3K related kinase.** The protein is involved in nonsense-mediated mRNA decay (NMD) as part of the mRNA surveillance complex. It has kinase activity and is thought to function in NMD by phosphorylating the regulator of nonsense transcripts 1 protein.	Kinase, RNA
USP4<br>0.032	**Ubiquitin specific peptidase 4.** This is a protease that deubiquitinates target proteins such as ADORA2A and TRIM21. The protein shuttles between the nucleus and cytoplasm and is involved in maintaining operational fidelity in the endoplasmic reticulum.	Protein metabolism, protease

Table S13. Comparison of the phosphoproteomic profile at the time of diagnosis for primary human AML cells derived from patients with many and few/no colony-forming cells. All protein information was obtained from genecards.org. Proteins that have been studied in AML are highlighted and PubMed Identifiers (PMIDs) are provided.

**Protein names, *p*-values**	**Phospho-sites**	**Description, increased in cultures ≥200 colonies**	**Keywords**
CAST (PMID: 22829235) 0.012	S11	**Calpastatin.** The protein is an endogenous calpain (calcium-dependent cysteine protease) inhibitor. The calpain/calpastatin system is involved in numerous membrane fusion events, such as neural vesicle exocytosis and platelet and red-cell aggregation. The protein is also thought to affect the expression levels of genes encoding structural or regulatory proteins.	Protease inhibitor
CPD 0.038	T1368 T1370	**Carboxypeptidase D** (see Table S11).	Transporter, carboxypeptidase
DOT1L (PMIDs: 24858818, 26972558, 27335278) 0.014	S902	**DOT1 like histone lysine methyltransferase.** The protein is a histone methyltransferase that methylates lysine-79 of histone H3. It is inactive against free core histones, but shows significant histone methyltransferase activity against nucleosomes.	Transcription factor, transferase
KIF4A 0.047	T799 S801	**Kinesin family member 4A.** This is an ATP dependent microtubule-based motor protein that is involved in the intracellular transport of membranous organelles. The protein also associates with condensed chromosome arms and may be involved in maintaining chromosome integrity during mitosis, and in the organization of the central spindle prior to cytokinesis.	Mitosis
PCBP1 (PMID: 26293996–retracted paper) 0.013	S246	**Poly(rC) binding protein 1.** A multifunctional protein. It, along with PCBP-2 and hnRNPK, corresponds to the major cellular poly(rC)-binding protein. Its three K-homologous domains may be involved in RNA binding. It also functions as translational coactivators of poliovirus RNA, human Papillomavirus type 16 L2 mRNA and hepatitis A virus RNA. The protein is also suggested to associate with α-globin mRNA stability.	mRNA splicing
SP100 0.022	S409	**SP100 nuclear antigen.** A subnuclear organelle and major component of the PML (promyelocytic leukemia) nuclear bodies. PML and SP100 are covalently modified by SUMO-1, which is considered crucial to nuclear body interactions. SP100 binds heterochromatin proteins and is thought to play a role in tumorigenesis, immunity, and gene regulation.	Chromatin binding
SPEN 0.035	S188	**Spen family transcriptional repressor.** A hormone-inducible transcriptional repressor. Repression of transcription can occur through interactions with other repressors, by the recruitment of proteins involved in histone deacetylation, or through sequestration of transcriptional activators. In addition, this repressor contains several RNA recognition motifs that confer binding to a steroid receptor RNA coactivator; this binding can modulate the activity of both liganded and non-liganded steroid receptors.	Transcription repressor
TCEAL3 0.028	S30	**Transcription elongation factor A like 3.** A member of the transcription elongation factor A (SII)-like (TCEAL) gene family. Members of this family may function as nuclear phosphoproteins that modulate transcription in a promoter context-dependent manner.	Transcription
VIM (PMID: 29925392)	S55	**Vimentin** (see Table S11).	(Cytoskeletal) signaling,

0.006			adhesion
ZFYVE26<br>0.037	S144	**Zinc finger FYVE-type containing 26.** The protein contains a FYVE zinc finger binding domain. The presence of this domain is thought to target these proteins to membrane lipids through interaction with phospholipids in the membrane.	(Cytoskeletal) signaling
ZNF521 (PMID: 28412727)<br>0.006	S605<br>S608	**Zinc finger protein 521.** Transcription factor that can both act as an activator or a repressor depending on the context. Involved in BMP signaling and in the regulation of the immature compartment of the hematopoietic system. Associates with SMADs in response to BMP2 leading to activate transcription of BMP target genes. Acts as a transcriptional repressor via its interaction with EBF1, a transcription factor involved specification of B-cell lineage; this interaction preventing EBF1 to bind DNA and activate target genes.	Transcription factor/repressor

**Protein names, *p*-values**	**Phospho-sites**	**Description, decreased in cultures ≥200 colonies**	**Keywords**
AEBP2 (PMID: 27022003)<br>0.014	S206	**AE binding protein 2.** DNA-binding transcriptional repressor.	Transcription repressor
ATP13A1<br>0.017	S899	**ATPase 13A1.** Mediates manganese transport into the endoplasmic reticulum. The ATPase activity is required for cellular manganese homeostasis.	(Ion) transporter
BOD1L1<br>0.013	S482<br>S484	**Biorientation of chromosomes in cell division 1 like 1.** Component of the fork protection machinery required to protect stalled/damaged replication forks from uncontrolled DNA2-dependent resection. Does not regulate spindle orientation.	DNA damage control
CAD<br>0.010	S1406	**Carbamoylphosphate synthetase 2, aspartate transcarbamylase and dihydroorotase.** The *de novo* synthesis of pyrimidine nucleotides is required for mammalian cells to proliferate. CAD is a trifunctional protein, which is associated with the enzymatic activities of the first 3 enzymes in the 6-step pathway of pyrimidine biosynthesis: carbamoyl-phosphate synthetase, aspartate transcarbamoylase, and dihydroorotase. This protein is regulated by MAPK, which indicates a direct link between activation of the MAPK cascade and *de novo* biosynthesis of pyrimidine nucleotides.	Pyrimidine biosynthesis
CFBF (PMID: 29192243)<br>0.008	S173	**Core-binding factor subunit β.** The β subunit of a heterodimeric core-binding transcription factor belonging to the PEBP2/CBF transcription factor family, which master-regulates a host of genes specific to hematopoiesis (e.g. RUNX1) and osteogenesis (e.g., RUNX2). The β subunit is a regulatory subunit as it allosterically enhances DNA binding by the α subunit. Pericentric inversion of chromosome 16 (creating the fusion protein CFBF-MYH11) is associated with AML FAB M4Eo.	Transcription factor, AML with inv(16)
CTPS1<br>0.014	S562	**CTP synthase 1.** An enzyme responsible for the catalytic conversion of UTP to CTP, which is an important step in the biosynthesis of phospholipids and nucleic acids.	Hydrolase, metabolism
DBNL<br>0.015	S269	**Drebrin-like.** Adapter protein that binds F-actin and dynamin 1, and thereby plays a role in receptor-mediated endocytosis. Plays a role in the reorganization of the actin cytoskeleton, formation of cell projections in neuron morphogenesis and synapse formation. Does not promote actin polymerization. May act as a common effector of antigen receptor-signaling pathways in leukocytes. Acts as a key component of the immunological synapse that regulates T-cell activation by bridging T-cell antigen receptors and the actin cytoskeleton to gene activation and endocytic processes.	Endocytosis, cytoskeleton
DNAJC2<br>0.005/0.006	S60<br>S63	**DnaJ heat shock protein family (HSP40) member C2.** A member of the M-phase phosphoprotein family. Contains both a J domain and a Myb DNA-binding domain, which localizes to both the nucleus and the cytosol. The protein is capable	Translation, stress response,

		of forming a heterodimeric complex that associates with ribosomes, acting as a molecular chaperone for nascent polypeptide chains as they exit the ribosome. This protein was identified as a leukemia-associated antigen and expression of the gene is upregulated in leukemic blasts.	chaperone
DNAJC5<br>0.027	S15	**DnaJ heat shock protein family (HSP40) member C5.** A member of the J protein family, which function in many cellular processes by regulating the ATPase activity of 70 kDa heat shock proteins. The protein plays a role in membrane trafficking and protein folding, and has been shown to have anti-neurodegenerative properties.	Chaperone, membrane trafficking
EIF5B<br>0.045	S107<br>S113	**Eukaryotic translation initiation factor 5B.** Factors eIF1A and eIF5B interact on the ribosome along with other initiation factors and GTP to position the initiation methionine tRNA on the start codon of the mRNA so that translation initiates accurately.	Translation initiation
FAM53C<br>0.033	S232<br>S234	**Family with sequence similarity 53 member C.** The FAM53 protein family members bind to a transcriptional regulator that modulates cell proliferation.	Proliferation modulation
GATAD2B<br>0.045	S134	**GATA zinc finger domain containing 2B.** A transcriptional repressor, which is part of the methyl-CpG-binding protein-1 complex that represses gene expression by deacetylating methylated nucleosomes.	Transcription repressor
GRAMD4<br>0.044	S24	**GRAM domain containing 4.** GRAMD4 is a mitochondrial effector of the transcription factor E2F1-induced apoptosis.	Mediates apoptosis
IRF2BP2<br>0.047	S441	**Interferon regulatory factor 2 binding protein 2.** The regulator interacts with the C-terminal transcriptional repression domain of IRF2.	Interferon regulator
ISL2<br>0.021	S154<br>S157	**ISL LIM homeobox 2.** Transcription factor that defines subclasses of motoneurons that segregate into columns in the spinal cord and select distinct axon pathways.	Transcription factor
LAT (PMID: 12540999—paper in Italian)<br>0.017/0.023	S40<br>S43	**Linker for activation of T-cells.** The protein is phosphorylated by Syk protein tyrosine kinase following activation of the T-cell antigen receptor signal transduction pathway. This transmembrane protein localizes to lipid rafts and acts as a docking site for SH2 domain-containing proteins. Upon phosphorylation, this protein recruits multiple adaptor proteins and downstream signaling molecules into multimolecular signaling complexes located near the site of T-cell antigen receptor engagement.	Signaling
LEO1 (PMIDs: 24686170, 30305722)<br>0.037	S140	**Leo 1 homolog.** Component of the PAF1 complex (PAF1C) which has multiple functions during transcription by RNA polymerase II and is implicated in regulation of development and maintenance of embryonic stem cell pluripotency. PAF1C associates with RNA polymerase II and is involved in transcriptional elongation. PAF1C is required for transcription of Hox and Wnt target genes. PAF1C is involved in hematopoiesis and stimulates transcriptional activity of KMT2A/MLL; it promotes leukemogenesis through association with MLL-rearranged oncoproteins.	Transcription, hematopoiesis
LSP1 (PMID: 29956722)<br>0.011/0.028/0.036	T175<br>S177	**Lymphocyte specific protein 1.** An intracellular F-actin binding protein, which is expressed in lymphocytes, neutrophils, macrophages, and endothelium and may regulate neutrophil motility, adhesion to fibrinogen matrix proteins, and transendothelial migration.	Signaling, chemotaxis
MAP1S (PMID: 25043887)<br>0.034	S703	**Microtubule associated protein 1S.** Mediates aggregation of mitochondria resulting in cell death and genomic destruction Plays a role in anchoring the microtubule organizing center to the centrosomes. Binds to DNA. Plays a role in apoptosis.	Apoptosis
MAP7D1<br>0.012/0.014	S113<br>S116	**MAP7 domain containing 1** (see Table S11).	Structural molecule?

MTOR (PMID: 25257528, 26985829) 0.045	S2454	**Mechanistic target of rapamycin.** Belongs to a family of phosphatidylinositol kinase-related kinases, which mediate cellular responses to stresses such as DNA damage and nutrient deprivation. The protein acts as the target for the cell-cycle arrest and immunosuppressive effects of the FKBP12-rapamycin complex. mTOR deregulation has been observed in many cancer types.	Signaling
MYO9B 0.012	S1972	**Myosin IXB.** A member of the myosin family of actin-based molecular motor heavy chain proteins. The protein represents an unconventional myosin. The protein binds calmodulin, which serves as a light chain. The protein complex has a single-headed structure and exhibits processive movement on actin filaments toward the minus-end. The protein also has rho-GTPase activity.	ATPase, cytoskeleton
MYSM1 0.023	S218	**Myb like SWIRM and MPN domains 1.** Metalloprotease that specifically deubiquitinates monoubiquitinated histone H2A, a specific tag for epigenetic transcriptional repression, thereby acting as a coactivator. Deubiquitination of histone H2A leads to facilitate the phosphorylation and dissociation of histone H1 from the nucleosome.	Transcription activator
NEK1 0.039	S983	**NIMA related kinase 1.** A serine/threonine kinase involved in cell cycle regulation. The protein is found in a centrosomal complex with FEZ1, a neuronal protein that plays a role in axonal development.	Kinase, cell cycle regulation
OSBP 0.035	S382	**Oxysterol binding protein.** Lipid transporter involved in lipid countertransport between the Golgi complex and membranes of the endoplasmic reticulum: specifically exchanges sterol with phosphatidylinositol 4-phosphate (PI4P), delivering sterol to the Golgi in exchange for PI4P, which is degraded in the endoplasmic reticulum.	Lipid transporter
PAPOLA 0.049	S537	**Poly(A) polymerase α.** Part of the poly(A) polymerase family. It is required for the addition of adenosine residues for the creation of the 3'-poly(A) tail of mRNAs.	pre-mRNA splicing
PPP6R3 0.015	S523	**Protein phosphatase 6 regulatory subunit 3.** Regulatory subunit of protein phosphatase 6. May function as a scaffolding subunit of the latter. May also have an important role in maintaining immune self-tolerance.	Phosphatase, metabolism
PRKD2 0.044	S206	**Protein kinase D2.** Belongs to the protein kinase D (PKD) family of serine/threonine protein kinases, which occupy a unique position in signaling pathways initiated by diacylglycerol and protein kinase C. PKDs are involved in cell survival, migration, differentiation and proliferation.	Kinase
PRPF31 0.012	S439	**Pre-mRNA processing factor 31**. Involved in pre-mRNA splicing as component of the spliceosome.	pre-mRNA splicing
PRPF40A 0.022	S888	**Pre-mRNA processing factor 40 homolog A.** Binds to WASL/N-WASP and suppresses its translocation from the nucleus to the cytoplasm, thereby inhibiting its cytoplasmic function (by similarity). Plays a role in the regulation of cell morphology and cytoskeletal organization. Required in the control of cell shape and migration. May play a role in cytokinesis, and in pre-mRNA splicing.	Cytoskeleton, pre-mRNA splicing?
RASGRP2 0.030	S116 S117	**RAS guanyl releasing protein 2.** Functions as a calcium- and diacylglycerol-G-regulated nucleotide exchange factor specifically activating Rap through the exchange of bound GDP for GTP. May also activates other GTPases such as RRAS, RRAS2, NRAS and KRAS. Functions in aggregation of platelets and adhesion of T-lymphocytes and neutrophils probably through inside-out integrin activation.	Calcium exchange, GTPase activator
RNF31 0.046	S466	**Ring finger protein 31.**The protein contains a RING finger, a motif present in a variety of functionally distinct proteins and known to be involved in protein-DNA and protein-protein interactions. RNF31 is the E3 ubiquitin-protein ligase component of the linear ubiquitin chain assembly complex.	Signaling, ligase
SCAP	S429	**SREBF chaperone.** This gene encodes a protein with a sterol sensing domain and seven WD domains. In the presence of	Lipid transporter

0.015		cholesterol, this protein binds to sterol regulatory element binding proteins and mediates their transport from the ER to the Golgi.	
SCRIB 0.031	S1306	**Scribbled planar cell polarity protein.** The mammalian protein is involved in tumor suppression pathways. As a scaffold protein involved in cell polarization processes, this protein binds to many other proteins.	Proliferation regulator
SH3BP1 0.033	S598	**SH3 domain binding protein 1.** A member of the rho GTPase activating protein family. The protein regulates Rac signaling and plays a role in cytoskeletal dynamics, cell motility and epithelial junction formation. Its association with the exocyst complex, which tethers secretory vesicles to the plasma membrane, has been demonstrated to be important in cell motility. In a distinct complex, this protein has been shown to regulate epithelial junction formation and morphogenesis.	Cytoskeleton, angiogenesis
TMPO 0.029	S66	**Thymopoietin.** The protein resides in the nucleus and may play a role in the assembly of the nuclear lamina, and thus help maintain the structural organization of the nuclear envelope. It may function as a receptor for the attachment of lamin filaments to the inner nuclear membrane.	(Cytoskeletal) signaling
TOR4A 0.003	S97	**Torsin family 4 member A.**	?
TTC7A 0.046	S51	**Tetratricopeptide repeat domain 7A.** Component of a complex required to localize phosphatidylinositol 4-kinase to the plasma membrane. The complex acts as a regulator of phosphatidylinositol 4-phosphate synthesis.	Regulating protein

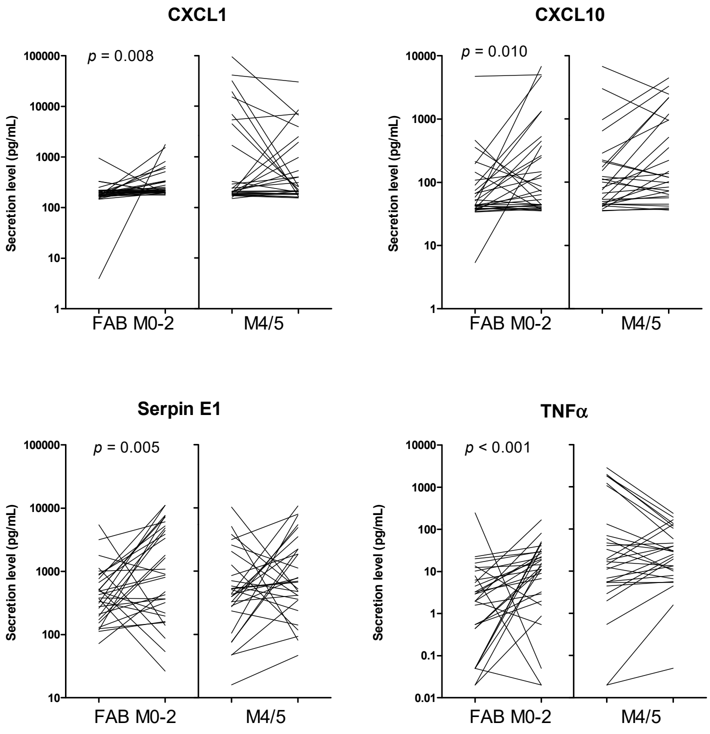

Figure S1. FAB M0-2 is associated with a relative concentration increase between weeks 1 and 5 for the mediators CXCL1, CXCL10, MMP-9, serpin E1 and TNF*α*. The figure also shows the wide concentration variations (factor 10^4 for MMP-9 and TNF*α*) among different patients, and that the initial (week 1) levels for CXCL1 and TNF*α* are much higher in cells with monocytic differentiation as compared to FAB M0-2.

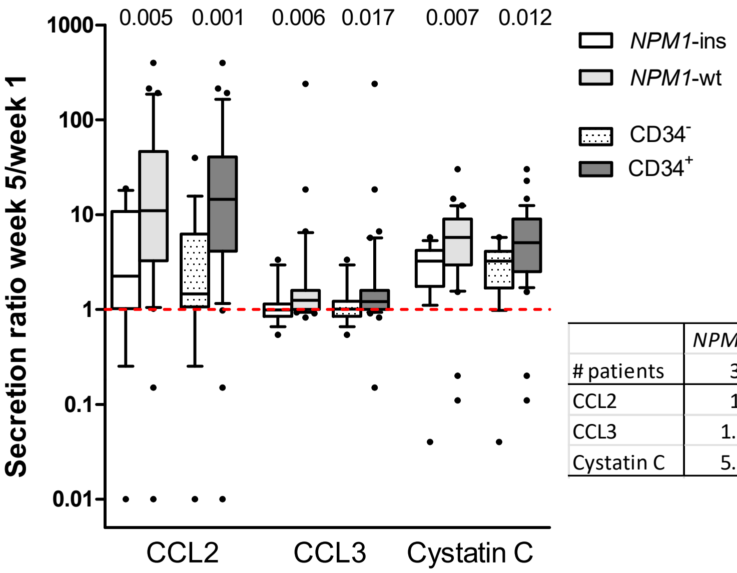

	NPM1-wt	*NPM1*-ins	CD34+	CD34-
# patients	31	18	39	18
CCL2	11	2.24	14.5	1.46
CCL3	1.26	0.99	1.21	1.01
Cystatin C	5.77	3.25	5.04	3.25

Figure S2. Increase in CCL2, CCL3 and cystatin C levels over time is associated with the *NPM1*-wt and $CD34^+$ patient subsets. The medium secretion ratios are provided in the table, whereas the *p*-values are given on top of the figure. There is a high degree of overlap as 14 patients present with both *NPM1*-ins and $CD34^-$. Furthermore, for four patients (2 with *NPM1*-ins and 2 with $CD34^-$), the status of the other parameter is unknown.

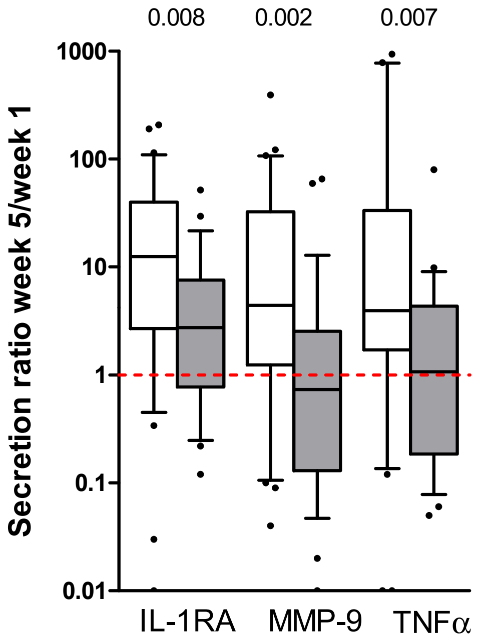

	FAB M0-2	FAB M4/5
# patients	30	28
IL-1RA	12.5	2.75
MMP-9	4.42	0.73
TNFα	3.95	1.07

Figure S3. Increase in IL-1RA, MMP-9 and TNF*α* levels over time is associated with the FAB M0-2 patient subset. The medium secretion ratios are provided in the table, whereas the *p*-values are given on top of the figure. Note that the MMP-9 ratios for the patient subset with more differentiated cells (FAB M4/5) decreases over time, whereas TNF*α* levels more or less remain unaltered.

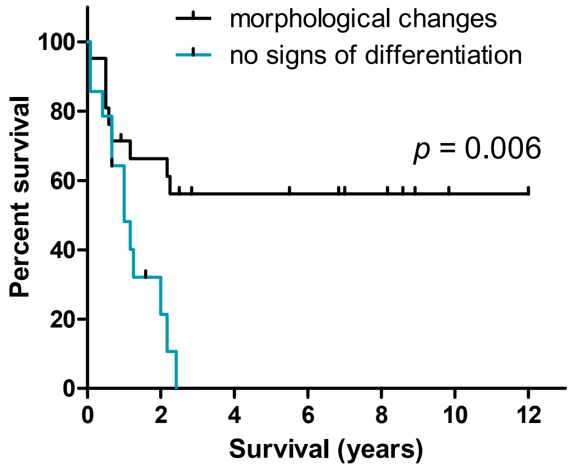

Variable	Crude HR	95% CI	*p*-value	Adj. HR	95% CI	*p*-value
Colony number (≥ 200)	3.24	1.30–8.06	**0.012**	3.38	1.32–8.67	**0.011**
Altered phenotype	0.30	0.12–0.76	**0.010**	0.29	0.12–0.74	**0.010**

Figure S4. Patient survival dependent on cell phenotype. The patients with cells that presented with changes in their phenotype during long-term culture (black line) showed significantly improved outcome (log-rank test) compared to patients with a stable cell phenotype. The hazard ratio calculation indicates that colony number and stable phenotype are independent risk factors in AML.

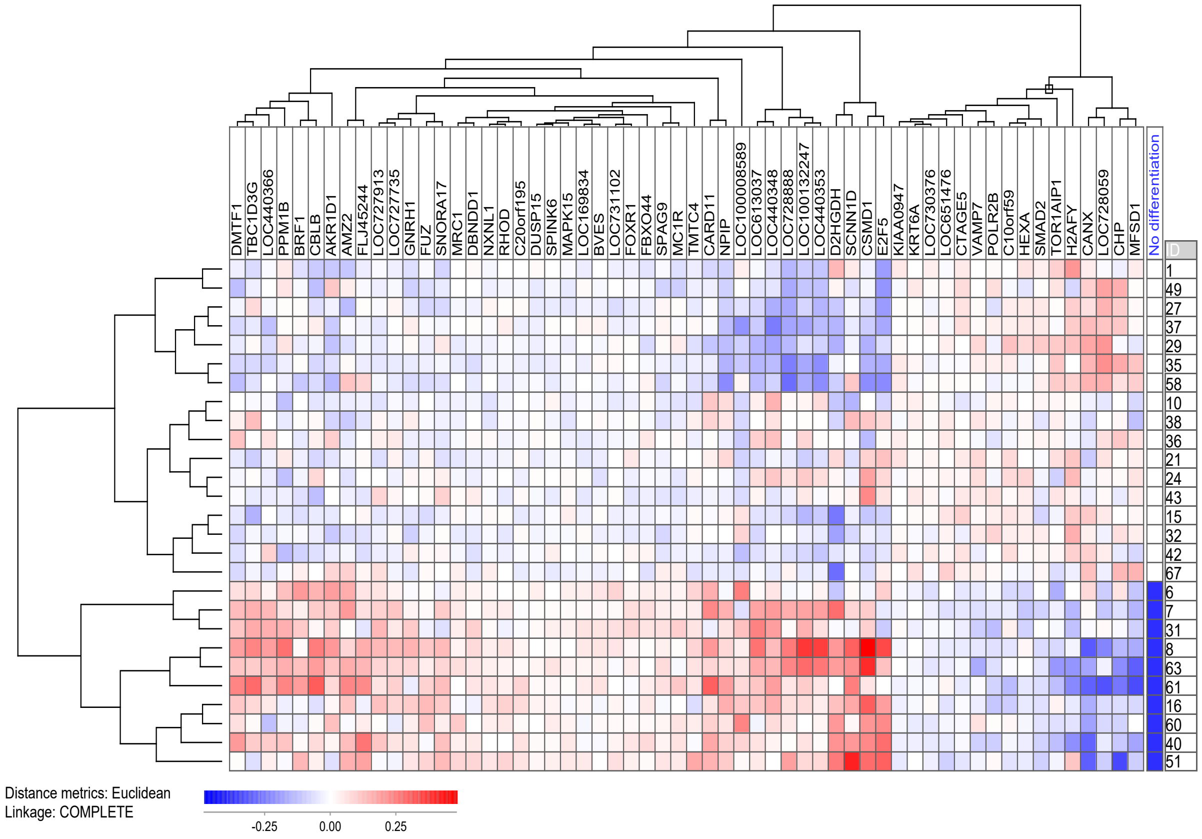

Figure S5. Cluster of the 58 differentially expressed genes between cultures with and without signs of morphological differentiation. Differentially expressed mRNA levels (t-score >4.00) were median normalized and $\log_{10}$ transformed prior to clustering. *CANX*, *DMTF1* and *NPIP* represent the mean value for two probes of the gene.

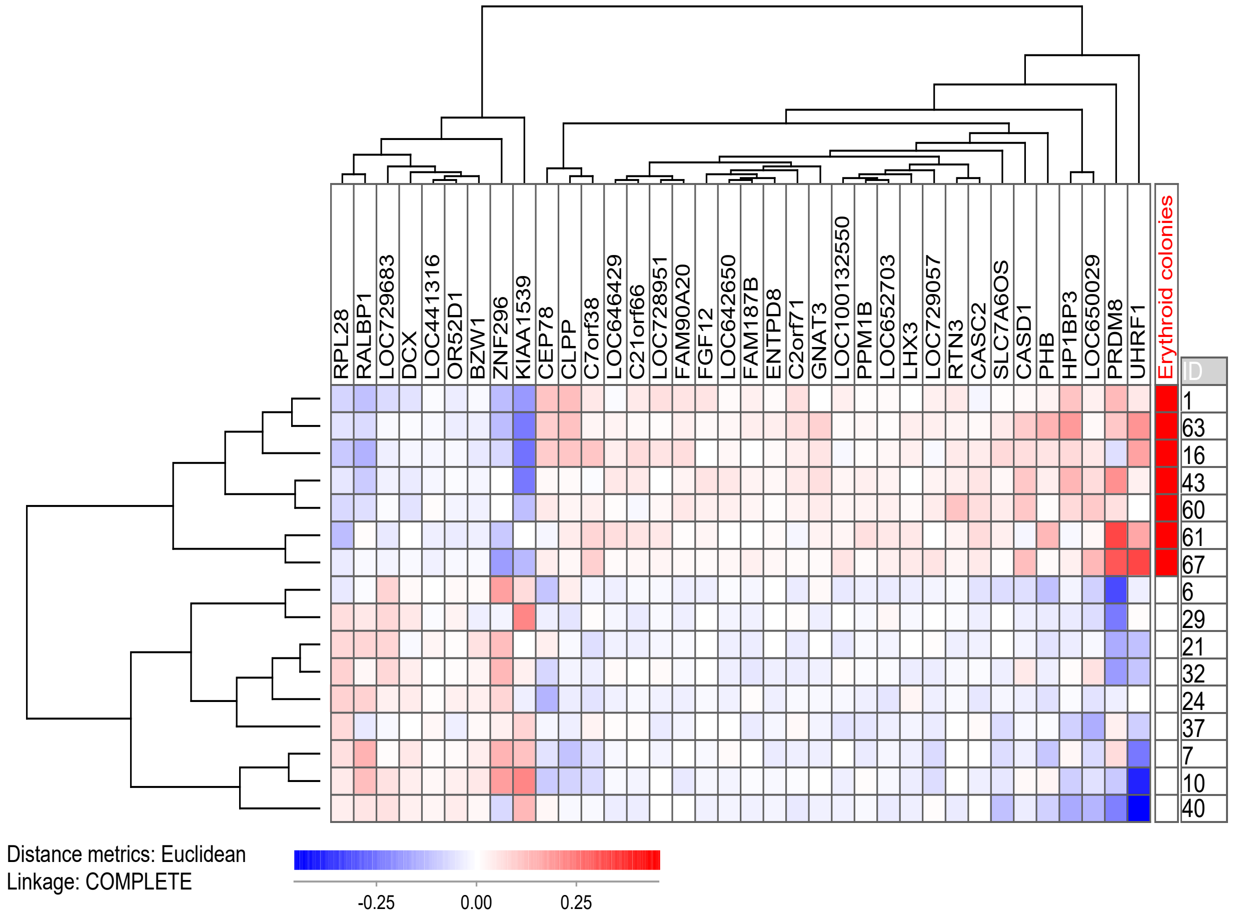

Figure S6. Cluster of the 36 differentially expressed genes between cultures with and without erythroid colonies. Differentially expressed mRNA levels (t-score >4.00) were median normalized and $\log_{10}$ transformed prior to clustering.
